# Supplementary figures and images for: Sex and gender differences in presentation, treatment and outcomes in acute coronary syndrome, a 10 year study from a multi-ethnic Asian population: The Malaysian National Cardiovascular Disease Database—Acute Coronary Syndrome (NCVD-ACS) registry
Source: PLoS One. 2021 Feb 8;16(2):e0246474. doi: 10.1371/journal.pone.0246474 (PMC7869989; doi:10.1371/journal.pone.0246474)

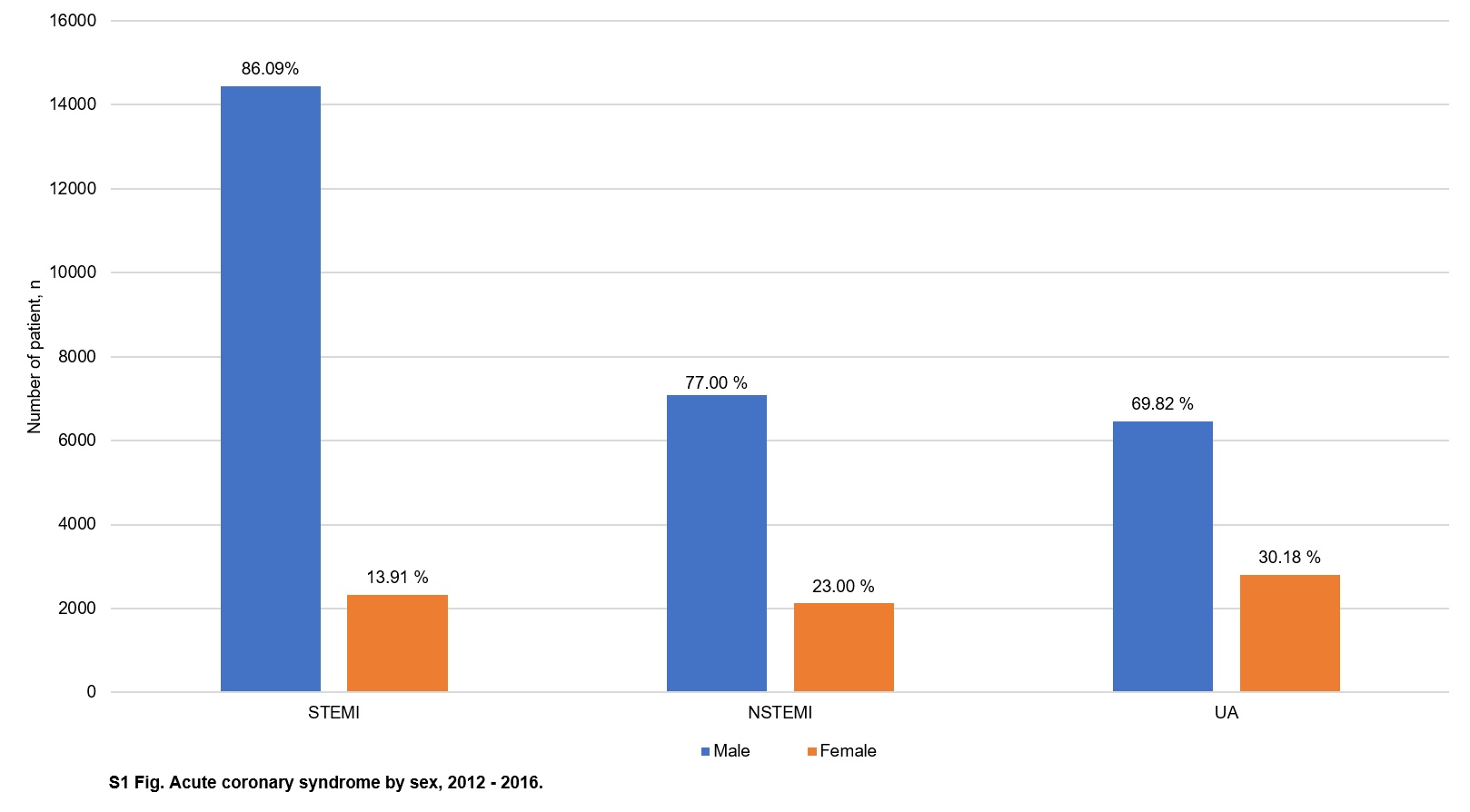

Supplement: S1 Fig — (TIF) [file pone.0246474.s001.tif]

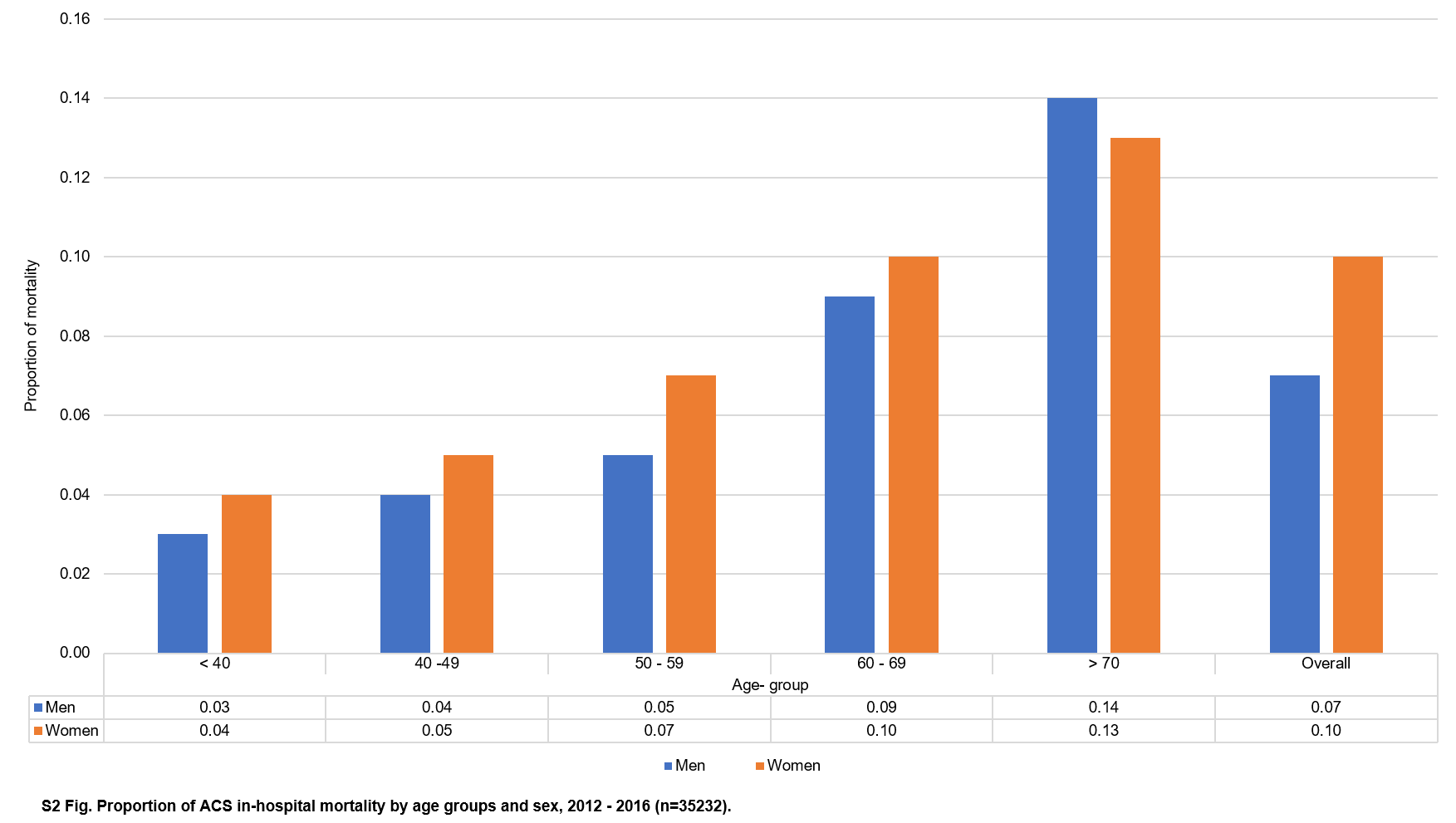

Supplement: S2 Fig — (TIF) [file pone.0246474.s002.tif]

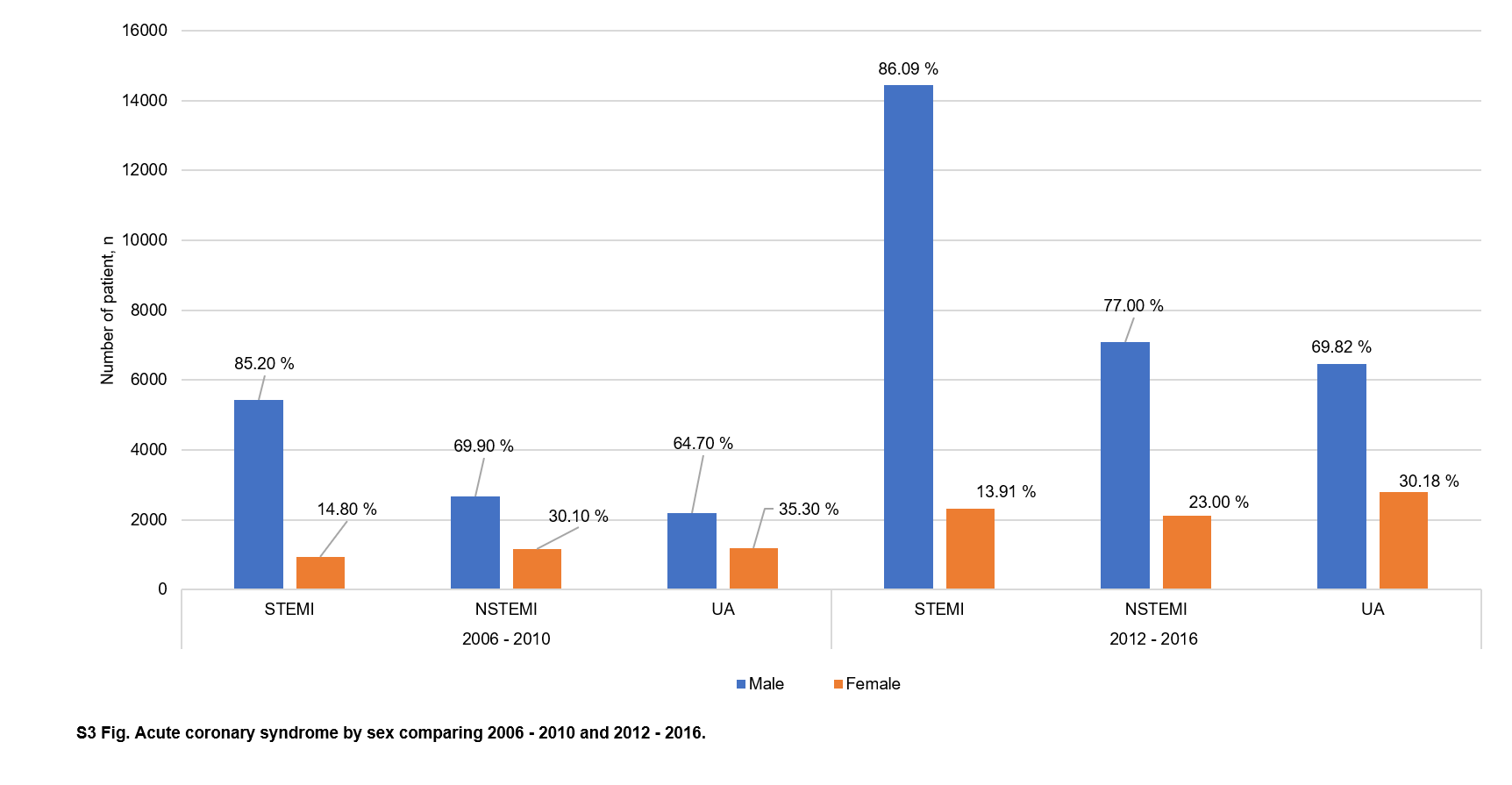

Supplement: S3 Fig — (TIF) [file pone.0246474.s003.tif]
