# Supplementary material for: Sex and gender differences in presentation, treatment and outcomes in acute coronary syndrome, a 10 year study from a multi-ethnic Asian population: The Malaysian National Cardiovascular Disease Database—Acute Coronary Syndrome (NCVD-ACS) registry
Source: PLoS One. 2021 Feb 8;16(2):e0246474. doi: 10.1371/journal.pone.0246474 (PMC7869989; doi:10.1371/journal.pone.0246474)
Supplement: S1 Table — (DOCX) [file pone.0246474.s004.docx]

**S1 Table. Treatment of STEMI, 2012- 2016.**

|  | Men | Women | Chi-square* | df | *p* value |
| --- | --- | --- | --- | --- | --- |
| Primary PCI † | 2039 (14.48) | 288 (12.97) | 73.56 | 2 | < 0.001 |
| Fibrinolysis † | 9830 (69.81) | 1422 (64.03) |  |  |  |
| No revascularization † ‡ | 2212 (15.71) | 511 (23.01) |  |  |  |
| Total † | 14081 (100.0) | 2221 (100.0) |  |  |  |
| Fibrinolysis subgroup |  |  |  |  |  |
| Type of fibrinolytic drug used † | |  |  |  |  |
| Streptokinase | 4840 (75.13) | 702 (75.65) | 0.12 | 1 | 0.734 |
| Other fibrinolytic drugs | 1602 (24.87) | 226 (24.35) |  |  |  |
| Door-to-needle time, ꝣ min | 45.0 (25.0, 90.0) | 59.0 (30.0, 120.0) |  |  | 0.001 ǁ |
| Door-to-balloon time, ꝣ min | 78.0 (49.0, 129.5) | 95.0 (56.0, 161.0) |  |  | 0.003 ǁ |

Values are n (%) or median (IQR).

df, degrees of freedom; IQR, interquartile range; other abbreviations as in Tables 1 and 3.

*Pearson chi-square.

†All categorical variables are expressed as n (%).

‡ Reason for no revascularization includes refusal, missed fibrinolysis, and contraindication.

ꝣMedian (IQR).

ǁ Mann-Whitney U test
