# Supplementary material for: Sex and gender differences in presentation, treatment and outcomes in acute coronary syndrome, a 10 year study from a multi-ethnic Asian population: The Malaysian National Cardiovascular Disease Database—Acute Coronary Syndrome (NCVD-ACS) registry
Source: PLoS One. 2021 Feb 8;16(2):e0246474. doi: 10.1371/journal.pone.0246474 (PMC7869989; doi:10.1371/journal.pone.0246474)
Supplement: S2 Table — (DOCX) [file pone.0246474.s005.docx]

**S2 Table. Prognostic factors for in-hospital mortality by sex, 2012-2016.**

|  | Male | | | | Female | | | |
| --- | --- | --- | --- | --- | --- | --- | --- | --- |
| Variables | n | Hazard ratio | 95 % CI | p value | n | Hazard ratio | 95% CI | p value |
| Age | nil | 1.03 | (1.02, 1.04) | < 0.001 | nil | 1.02 | (1.00, 1.04) | 0.034 |
| Heart rate | nil | 1.02 | (1.01, 1.02) | < 0.001 | nil |  |  |  |
| Admission SBP | nil | 0.99 | (0.99, 1.00) | 0.012 | nil |  |  |  |
| Killip class IV at presentation |  |  |  |  |  |  |  |  |
| I, II, III | 19715 | 1 |  |  | 4573 | 1 |  |  |
| IV | 2468 | 2.71 | (2.05, 3.58) | < 0.001 | 597 | 5.87 | (3.76, 9.16) | < 0.001 |
| Elevated creatinine kinase |  |  |  |  |  |  |  |  |
| Normal | 10363 | 1 |  |  | 3854 | 1 |  |  |
| Elevated creatinine kinase | 13008 | 1.71 | (1.23, 2.35) | 0.001 | 2253 | 1.93 | (1.19, 3.11) | 0.007 |
| PCI |  |  |  |  |  |  |  |  |
| No | 17697 | 1 |  |  | 5347 | nil |  |  |
| Yes | 8005 | 0.7 | (0.53, 0.92) | 0.01 | 1325 |  |  |  |
| Aspirin |  |  |  |  |  |  |  |  |
| No | 902 | nil |  |  | 315 | nil |  |  |
| Yes | 26476 | 0.49 | (0.28, 0.86) | 0.013 | 6754 |  |  |  |
| Beta-blockers |  |  |  |  |  |  |  |  |
| No | 9592 | 1 |  |  | 2639 | 1 |  |  |
| Yes | 16025 | 0.29 | (0.21, 0.41) | < 0.001 | 4072 | 0.56 | (0.34, 0.94) | 0.029 |
| ACE-I |  |  |  |  |  |  |  |  |
| No | 12326 | 1 |  |  | 3410 | 1 |  |  |
| Yes | 13088 | 0.33 | (0.22, 0.50) | < 0.001 | 3214 | 0.23 | (0.10, 0.52) | < 0.001 |
| Statins |  |  |  |  |  |  |  |  |
| No | 1931 | 1 |  |  | 577 | 1 |  |  |
| Yes | 25138 | 0.51 | (0.36, 0.71) | < 0.001 | 6417 | 0.44 | (0.26, 0.72) | 0.001 |

Backward stepwise Cox proportional hazards regression model applied
