# Supplementary material for: Sex and gender differences in presentation, treatment and outcomes in acute coronary syndrome, a 10 year study from a multi-ethnic Asian population: The Malaysian National Cardiovascular Disease Database—Acute Coronary Syndrome (NCVD-ACS) registry
Source: PLoS One. 2021 Feb 8;16(2):e0246474. doi: 10.1371/journal.pone.0246474 (PMC7869989; doi:10.1371/journal.pone.0246474)
Supplement: Attachment — Submitted filename: Response to Reviewers.docx [file pone.0246474.s006.docx]

**Response to Reviewers**

Dear Editor and reviewers,

Thank you for your kind comments. Following are my response to the queries raised:

1) During the writing of the first manuscript, Sex differences in acute coronary syndrome in a multi-ethnic Asian population: Results of the Malaysian National Cardiovascular Disease Database-Acute Coronary Syndrome (NCVD-ACS) Registry, February 2010 was the latest available clean data. Likewise, during the writing of the present manuscript December 2016 was the latest available clean data. As we wanted to compare two equivalent time period, we therefore choose 2012 to 2016. The data from March 2010 to Dec 2011 are available in the Annual Report of the NCVD-ACS Registry published and available online at <http://www.acrm.org.my/ncvd>.
2) Statistical analyses comparing women and men on the various parameters were done for the first cohort from 2006 to 2010 and second cohort from 2012 to 2016 as described in the methods section. As my aim was to compare these two periods to see if there is any change in trends in the patients’ characteristics, clinical presentations, treatments and outcomes, no further statistical analysis were done. To enable readers to see the trend easily, we provide multiple bar charts with the actual value of the variables shown at the bottom of the chart for the more avid readers who prefer to read numbers.
3) Additional requirements:
i) I have checked to ensure the manuscript meets PLOS ONE’s style requirements.
ii) Regarding the authors list, I am the fisrt author and the other 5 co-authors are as listed. There is no group involved in the writing of this manuscript. I wanted to acknowledge the principal investigators from each of the centers that contributed raw data to the NCVD-ACS registry but did not fulfil authorship criteria. Since they do not meet authorship requirement I will mention their names in the acknowledgement section as suggested. My apologies for the mistake.

Thank you.

Sincerely,

Dr Chuey Yan Lee.
